# *Froriepia subpinnata* Leaf Extract-Induced Apoptosis in the MCF-7 Breast Cancer Cell Line by Increasing Intracellular Oxidative Stress

**DOI:** 10.5812/ijpr-136643

**Published:** 2023-10-14

**Authors:** Hanieh Rostamabadi, Mohammad Rasoul Samandari Bahraseman, Keyvan Esmaeilzadeh-Salestani

**Affiliations:** 1 Department of Biology, Faculty of Sciences, Shahid Bahonar University of Kerman, Kerman, Iran; 2 Plant Production and Genetic Engineering Department, Faculty of Agriculture, Lorestan University, Khorramabad, Iran; 3 Varjavand Kesht Kariman, Limited Liability Company, Kerman, Iran; 4 Department of Biotechnology, Faculty of Science and Modern Technology, Graduate University of Advanced Technology, Kerman, Iran; 5 Chair of Crop Science and Plant Biology, Institute of Agricultural and Environmental Sciences, Estonian University of Life Sciences, Kreutzwaldi 1, EE51014 Tartu, Estonia

**Keywords:** *Froriepia subpinnata*, Apoptosis, Intracellular Oxidative Stress, Breast Cancer

## Abstract

**Background:**

*Froriepia subpinnata* is one of the plants used in the diet of Iranian people. Previous studies have investigated the antioxidant and antibacterial effects of this plant extract, but no study has been conducted on its anticancer properties.

**Objectives:**

In this study, we investigated the effect of *F. subpinnata* extract on MCF-7 breast cancer cells.

**Methods:**

The inhibitory effect of *F. subpinnata* leaf extract was determined on the growth of cancer cells by the MTT test. The ROS (reactive oxygen species) test was used to investigate the impact of the extract on intracellular oxidative stress. Flow cytometry and real-time PCR tests were used to investigate the apoptosis-related molecular processes. The GC-MS analysis was performed to determine the most abundant components.

**Results:**

The GC-MS analysis showed that phytol, mono-ethylhexyl phthalate (MEHP), cinnamaldehyde, and neophytadiene constituted 60% of the extracted content. The MTT assay demonstrated that *F. subpinnata* leaf extract caused 50% lethality at a 400 μg/mL dose in MCF7 cells. The *F. subpinnata* extract at low doses decreased the ROS level for 24 hours in MCF-7, but by increasing the concentration, the ROS levels increased. At the IC_50_ dose (inhibitory concentration (IC) associated with 50% impact), the ROS level increased 3.5 times compared to the control group. Examining the effect of N-acetyl cysteine (NAC) showed that this antioxidant agent could prevent the lethal impact of the extract and eliminate the ROS increase in MCF7 cells. Flow cytometry and real-time PCR results showed that the extract specifically induced apoptosis through the internal apoptosis pathway in this cancer cell line.

**Conclusions:**

The *F. subpinnata* extract induced apoptosis by increasing ROS in MCF-7 cancer cells and can be considered for further studies.

## 1. Background

Breast cancer is the most common malignancy worldwide and the main cause of cancer-related deaths. The issue of breast cancer treatment has received considerable critical attention (1). Breast cancer has become a fatal threat to women's health (2). Cancer prevention and treatment can coexist with traditional approaches. Indeed, numerous tests on breast cancer cell survival and proliferation revealed strong additive or synergistic effects of combinations of anticancer drugs/extracts from a plant that may decrease the unspecific toxicity of anticancer drugs (3). Since chemotherapeutic agents and cancer treatment methods are not very effective in cancer therapy, research on medications or new methods that affect apoptosis resistance has become more practical (4, 5).

Tumor expansion or regression is based on the balance between apoptosis and cell proliferation. Programmed cell death, which is called apoptosis, is a programmed process of cell suicide in response to specific signals, both extracellular and intracellular. Apoptosis is genetically or chemically regulated under certain conditions, such as cancer and autoimmune diseases. The ratio between apoptosis and cell proliferation controls normal growth. On the other hand, intracellular challenges such as reactive oxygen species (ROS), hypoxia, and nutrient deprivation can disrupt homeostasis regulation (6, 7). In this regard, an excessive increase in ROS levels can induce apoptosis (8, 9). Although it has been revealed in various studies that plant extracts have antioxidant properties (10-12), many studies have shown that plant extracts could increase ROS in cancer cells, which ultimately leads to apoptosis (13-15).

Investigating natural foods and their derived compounds is a continuous concern in medical research to treat cancer. Several types of papers have been conducted about chemopreventive compounds against cancers. Understanding the mutual link between natural products and apoptosis in cancer can lead to better medicines that are less toxic and act more specifically. Previous studies have indicated that natural compounds not only induced apoptosis but also reduced chemotherapy resistance (6, 16-18).

Since medicinal plants can stop the growth and development of tumor cells without serious damage to normal cells, they could be suitable medicines for cancer therapy (19-21). *Froriepia subpinnata* (*F. subpinnata*) is a medicinal plant from the Apiaceae family and native to northern Iran, which also has different species in Eurasia and Africa (22). Because of its valuable chemical compounds, the Apiaceae family has antioxidative, anticancer, antibacterial, and anti-inflammatory properties (23, 24). *Froriepia subpinnata* has chemical compounds such as triterpenoid, saponins, flavonoids, and coumarins that help human health as an appetizer, antiseptic, antispasmodic, sedative, and anti-allergy agent (25, 26). Since this plant has antibacterial properties, it is suggested to be used in the pharmaceutical and food industries (27, 28). The most abundant compounds of this plant are p-cymen-8-ol, α-Terpinolene, limonene, E-ocimenone, neophytadiene, and β-elemene, in sequence (25).

## 2. Objectives

As mentioned before, breast cancer is one of the fatal diseases. Hence, this study aimed to determine the impact of *F. subpinnata* extract on apoptotic activity and its anticancer properties on breast cancer cell line MCF-7.

## 3. Methods

### 3.1. Preparation of Froriepia subpinnata Leaf Extract

To prepare *F. subpinnata* leaf methanolic extract, the leaves were dried in the shade at room temperature (22 - 25°C). Then, they were powdered by an electric mill. Next, the obtained powder was mixed with methanol (80%) and shaken for 48 hours at room temperature. Finally, to obtain a clear solution, the extract was refined several times with Whatman filter paper (Grade 1), and then the organic solvent was concentrated via a rotary evaporator at 40°C.

### 3.2. Essential Oil Extraction and GC-MS Analysis

The essential oil was prepared using the Clevenger method. Then, GC-MS was performed using helium gas at a 1 mL/min flow rate, and the injector temperature was set at 250°C. After maintaining a temperature of 50°C for 2 minutes, the oven temperature gradually increased at a rate of 10°C per minute. After 15 minutes, the final temperature reached 250°C. The GC-MS analysis was conducted using a Thermo Finnigan Trace DSQ GC-MS machine. A DB-5 capillary column, equipped with helium as the carrier gas, was used to operate it at a 70 eV ionization energy. We determined the constituents by comparing their relative retention time (RT) and mass spectra to those of standards, NIST, Wiley, and Adams library data of the GC-MS system, as well as literary sources.

### 3.3. Cell Culture and MTT Assay

The MCF-7 cell line was purchased from the Iranian Genetic Resources Center in Tehran, Iran. For the proliferation of MCF-7 cells, DMEM media containing 10% FBS and 1% antibiotics were used, while MCF-10A cells were cultured in RPMI. The incubation of cells was done at 37°C, in 5% CO_2_, with 95% humidity. To evaluate the viability of cells treated with the extract, the MTT assay was used. For this purpose, 5000 cells/well of the MCF-7 cell line were cultured in a 96-well plate and then incubated for 24 h. After that, *F. subpinnata* in different concentrations was applied to treat the cells. After 24 h treatment, the first 30 μL of the MTT solution (0.5 μg/mL in PBS) was added to each well, and then the plates were incubated for 3 h. After the culture medium was removed, 100 μL of DMSO was added to each well, and then the plate was gently shaken. Finally, the percentage of cell viability was calculated by reading the absorbance of each well at 570 nm by an ELISA reader (Eliza MAT 2000, DRG Instruments, GmbH, Marburg, Germany). The 5 mM concentration of NAC (N-acetyl cysteine) was used for the NAC and combined groups, and the solvent of both the extract and NAC was the culture medium.

### 3.4. Intracellular ROS Assay

The amount of ROS was measured by the ROS assay Kit (Teb Pazhouhan Razi Company) based on the manufacturer's instructions. Briefly, as the cultured cells in a 96-well plate reached 80% confluency, they were treated with the intended concentrations of the extract. After 24 h, the cell culture medium was removed, and then 100 μL of ready assay buffer was added to each well. Next, the buffer was removed, and 100 μL of DCF staining buffer was added. After the plate was held at 37°C in the dark for 60 min, 100 μL of stimulator buffer was added, and the plate was kept at 37°C for 20 min in the dark. Eventually, the wells were emptied, 100 μL of the ready assay buffer was added, and the plate was read at the excitation wavelength of 485 nm and the emission wavelength of 538 nm by a plate reader (Eliza MAT 2000, DRG Instruments, GmbH, Marburg, Germany).

### 3.5. Gene Expression Assessment

To assess the effect of the compounds on the expression of *BAX*, *BCL-2*, and *TP53* genes, real-time PCR was performed. In summary, RNA was initially extracted from treated cells with TRIzol reagent according to the manufacturer's protocol. For measuring the quality and concentration of the extracted RNA, NanoDrop™ 2000/2000c spectrophotometer was used. Next, cDNA was synthesized using a Thermo Scientific Kit. Next, using RealQ Plus 2x Master Mix (AMPLIQON), real-time PCR was accomplished. The *GAPDH* gene was utilized as a housekeeping gene. The primers were designed for each gene and controlled for their specificity using BLAST on the NCBI site. The 2^-ΔΔCT^ formula was used to calculate the percentage of the relative gene expression. The primers used in this study are as follows: *GAPDH* forward primer: '5CCCCAGCAAGAGCACAAGAGG3' and reverse primer: '5-AGGAGGGGAGATTCAGTGTGG 3'; *BCL-2* forward primer: 5'TGGGGTCATGTGTGTGGA-G3' and reverse primer: 5'CGGTTCAGGTACTCAGTCATCC3'; *BAX* forward primer: 5'CC-CGAGAGGTCTTTTTCCGAG3' and reverse primer: 5'CCAGCCCATGATGGTTCTGAT3'; *P53* forward primer: 5'ACCTAAAAGGAAATCTCACCC3' and reverse primer: 5'ACCCTGAG-CATAAAACAAGTC3'.

### 3.6. Flow Cytometry Analysis

The apoptosis and necrosis in the cells exposed to the extract were studied by the Annexin-V PE/7-ADD apoptosis diagnosis kit according to the manufacturer's guidelines. Briefly, the cells were treated with the extract for 24 h, then 60,000 cells were washed with PBS and suspended in 100 μL of Annexin V binding buffer, 5 μL of PE Annexin V, and 5 μL of 7-AAD (7-amino actinomycin D). Finally, after keeping them in the dark for 15 min, 400 μL of binding buffer was added, and samples were assayed with a flow cytometer (Becton Dickinson, Franklin Lakes, NJ).

### 3.7. Statistical Analysis

We used SPSS software to analyze the data. Analysis of variance (ANOVA) and the Tukey test were used to estimate the significant differences among the groups. All experiments were repeated three times. The results were presented as mean ± SEM. Also, P < 0.05, P < 0.01, and P < 0.001 were defined as statistically significant levels.

## 4. Results

### 4.1. Froriepia subpinnata GC-MS Results

The GC-MS analysis of *F. subpinnata* leaf extract was done to specify the bioactive compounds. The GC-MS chromatogram (Table 1) showed that *F. subpinnata* comprised 32 compounds. The most abundant compounds of this plant extract, comprising 60% of the content, were phytol, mono-ethylhexyl phthalate (MEHP), cinnamaldehyde, and neophytadiene. Other identified compounds, each comprising less than 2% of *F. subpinnata*, were Alpha limonene, dodecane, 2-pentanone 4-hydroxy 4-methyl, tetradecane, 1-chlorooctadecane, N-hexadecyl bromide, tetra methyl adamantane, neophytadiene (isomer), phytol acetate, octadecane, hexahydro pseudoionone, eugenol, nonadecane, anozol, phthalic acid, ethyl isopropyl ester, citric acid, triethyl ester, pentacosane, hexacosane, heptacosane, guanosine, and leucoglucosan.

**Table 1.** GC-MS Analysis of *Froriepia subpinnata* Leaf Extract ^a^

Number	Compounds	RT (min)	Percentage
**1**	Phytol	16.99	28.89
**2**	Mono-ethylhexyl phthalate	20.97	14.02
**3**	Cinnamaldehyde	11.91	9.69
**4**	Neophytadiene	10.85	5.59
**5**	Docosane	16.05	4.67
**6**	Henicosane	15.22	3.36
**7**	Tricosane	16.86	3.3
**8**	Pentadecane	9.44	2.75
**9**	Tetracosane	17.64	2.7
**10**	Eicosane	14.36	2.63
**11**	Hexadecane	10.54	2.37

^a^All Compounds, Each Comprising More Than 2% of *F. Subpinnata*, are Mentioned in the Table.

### 4.2. Froriepia subpinnata Inhibited the Viability of MCF-7 and MCF-10A Cell Lines

**Figure 1.**
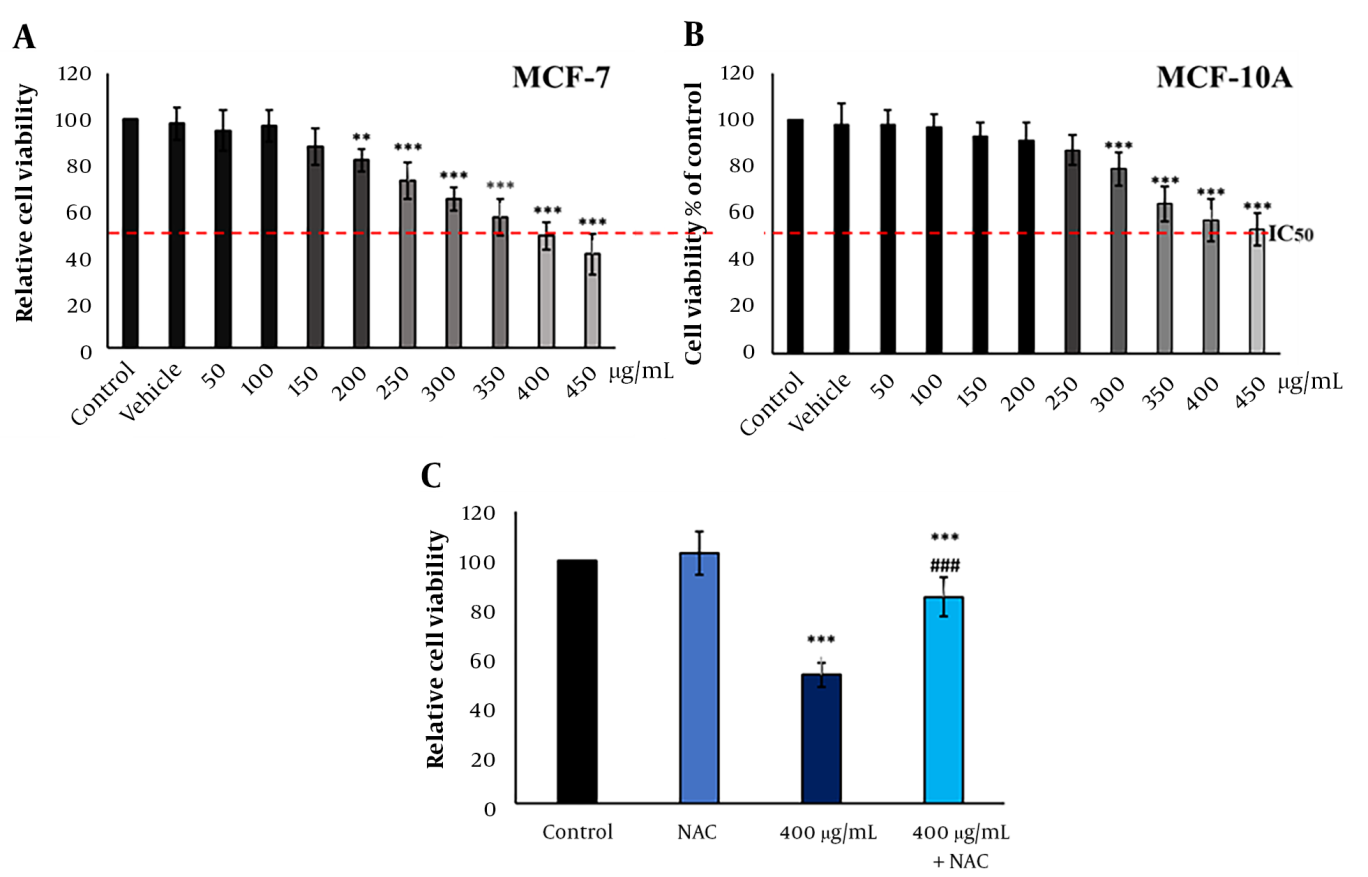
The effect of *F. subpinnata* extract on the viability of MCF-7 cells. (A) The effect of different extract concentrations on MCF-7 cell viability after 24 hours. (B) The effect of *F. subpinnata* extract on the viability of MCF-10A cells. (C) The combination effect of extract (IC_50_) with NAC on MCF-7 cell viability after 24 hours. ** and *** illustrate significant differences compared to the control with P < 0.01 and 0.001, respectively. ### indicates a significant difference compared to the IC_50_ group at P < 0.001. The experiments were done thrice, and the results are presented as mean ± SD.

*Froriepia subpinnata* extract declined the viability of MCF-7 and MCF-10A cell lines in a dose-dependent manner. The IC_50_ of the extract for MCF-7 cells was about 400 μg/mL. The extract did not significantly affect cell viability up to 200 μg/mL compared to the control group (Figure 1A). Moreover, MCF-10A cells treated with the extract showed more resistance to cell death than the control group, and no meaningful effect on cell viability was seen until the dose of 300 μg/mL. The IC_50_ was about 450 μg/mL (Figure 1B). 

Compared to the IC_50_ group, the combination of NAC and IC_50_ (combined group) significantly eliminated the inhibitory effect of the extract on the viability rate. However, the combined group had a remarkably lower survival rate than the control group. The NAC group did not have a clear change compared to the control group (Figure 1C). 

### 4.3. Froriepia subpinnata Increased the ROS Levels of MCF-7 Cells

**Figure 2.**
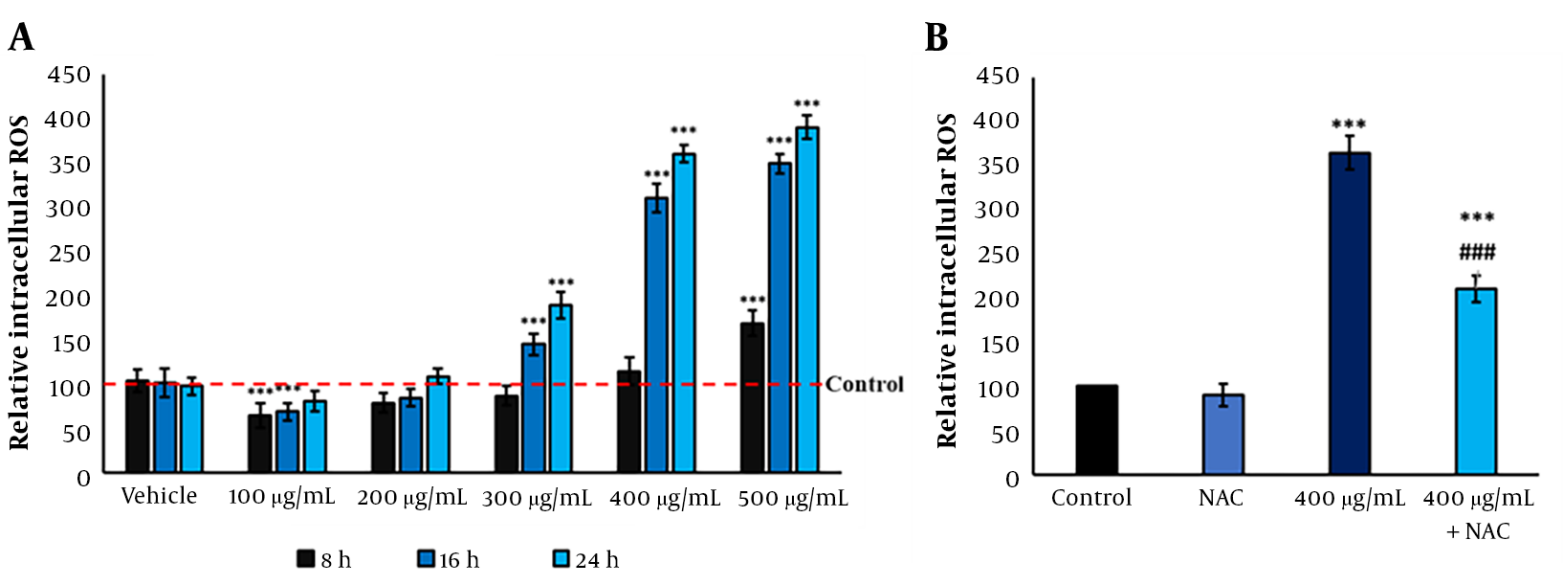
The ROS levels after treatment with *F. subpinnata*. (A) The effect of the extract on ROS level after 8, 16, and 24 hours in different concentrations. (B) The effect of IC_50_ and combined group on the ROS levels after 24 hours. * and *** show significant differences compared to the control with P < 0.05 and 0.001, respectively. ### indicates a significant difference compared to the IC_50_ group at P < 0.001. The experiments were done thrice, and the results are presented as mean ± SD.

Compared to the control group, *F. subpinnata* extract significantly increased the ROS levels in MCF-7 cells from the dose of 300 μg/mL onwards, depending on the duration and concentration. The IC50 induced a 3.5-time increase in the ROS level compared to the control after 24 hours. On the other hand, the 200 μg/mL dose did not noticeably affect the level of ROS; even the dose of 100 μg/mL alleviated the intracellular ROS level (Figure 2A). Although the combined group reduced the ROS level compared to the IC50, it showed a significantly higher ROS level than the control group (Figure 2B). 

### 4.4. Froriepia subpinnata-Induced Apoptosis in MCF-7 Cells

**Figure 3.**
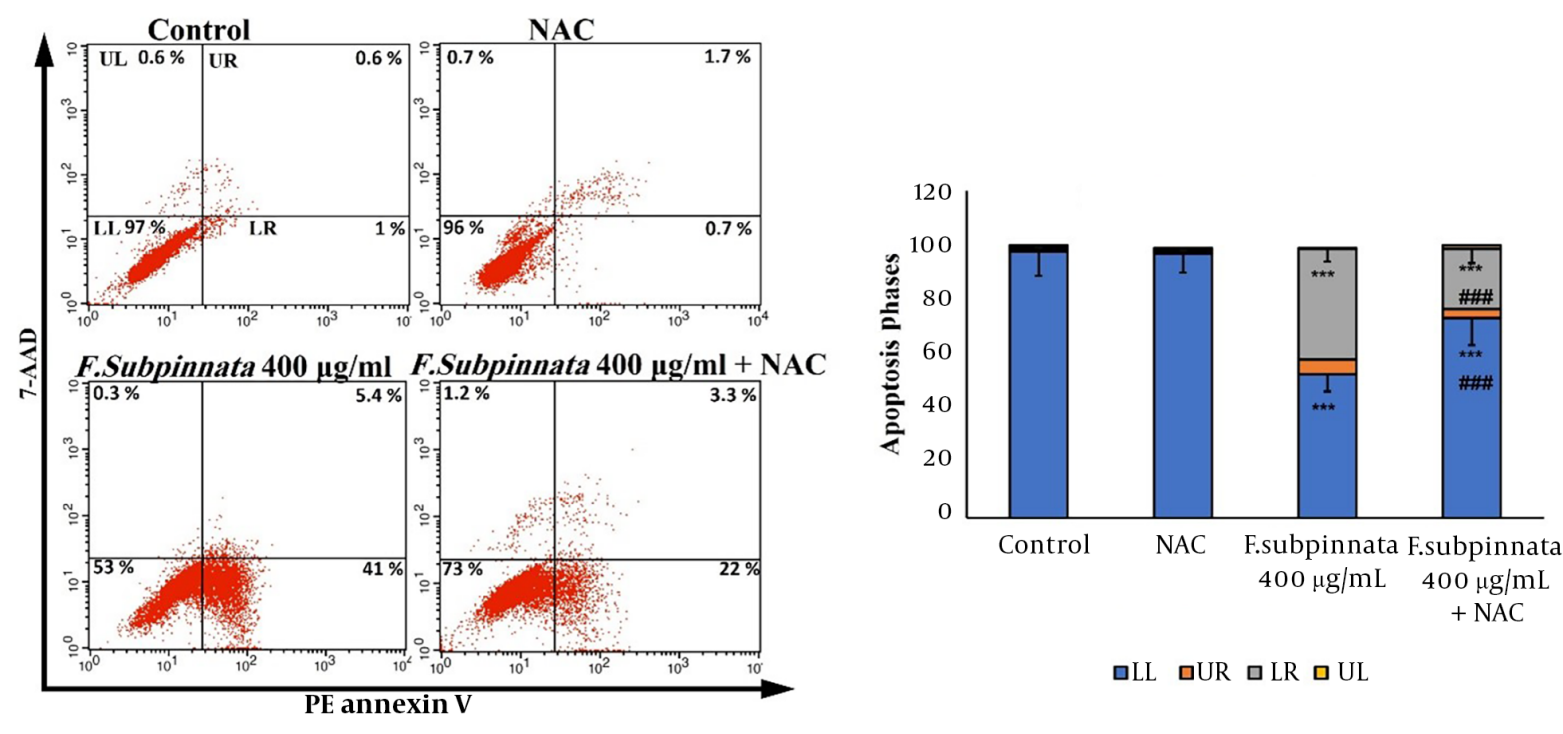
The flow cytometry results of the apoptotic effect of *F. subpinnata* extract and its combination with NAC on MCF-7 cells for 24 hours. UL: necrosis, LR: primary apoptosis, UR: late apoptosis, LL: living cells. *** shows significant differences compared to the control with P < 0.001. ### indicates a significant difference compared to the IC_50_ group at P < 0.001. The experiments were done three times, and the results are presented as mean ± SD.

The flow cytometry results indicated that the extract at the dose of IC_50_ caused apoptosis in MCF-7 cells compared to the control group. The combined group eliminated apoptosis compared to the IC_50_ group. Despite that, apoptosis was observed in the combined group compared to the control group (Figure 3). 

### 4.5. Froriepia subpinnata Modulated Gene Expressions Related to Apoptosis in MCF-7 Cells

The expression of gene *TP53* in the IC_50_ group was significantly higher compared to the control group. Although the expression level of this gene was lower in the combined group than in the IC_50_ group, it was remarkably higher compared to the control group.

A significant increase in *BAX* expression was observed in the IC_50_ group compared to the control group. The *BAX* expression showed lower expression in the combined group than in the IC_50_ group, while the *BAX* expression had a clear increase compared to the control group.

**Figure 4.**
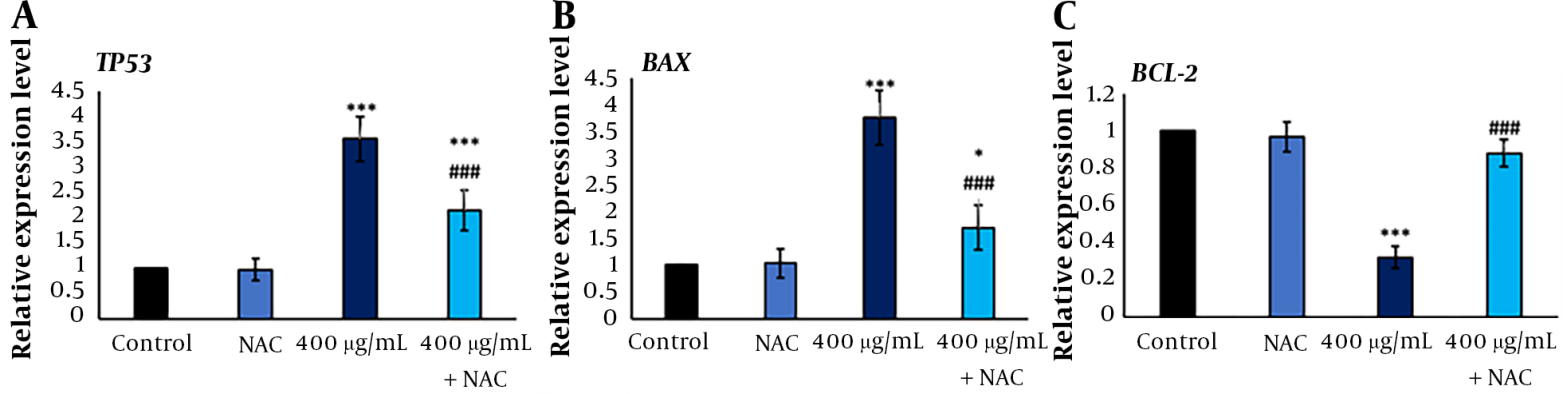
The *TP53*, *BAX*, and *BCL-2* gene expression levels after MCF-7 treatment with *F. subpinnata* (IC_50_ and combination of NAC and IC_50_) after 24 hours. * and *** show significant differences compared to the control with P < 0.05 and 0.001, respectively. ### indicates a significant difference compared to the IC_50_ group at P < 0.001. The experiments were done three times, and the results are presented as mean ± SD.

*BCL-2* expression was noticeably lower in the IC_50_ group than in the control group. The expression of this gene was higher in the combined group than in the IC_50_ group, and it did not have a significant decrease compared to the control group (Figure 4). 

## 5. Discussion

According to the World Health Organization, cancer is one of the main causes of death worldwide. In 2020, it caused about 10 million deaths, and 2.26 million cases were due to breast cancer (29). As mentioned in previous studies, many plant extracts have been identified as effective anticancer compounds (30, 31). The Apiaceae family is a large family with more than 3,000 species worldwide. The plants of this family are famous for their medicinal properties, such as estrogenic, anti-inflammatory, anticancer, antiproliferative, cytotoxic, antimicrobial, and anti-neoplastic effects. Since Apiaceae are a very important source of phytochemicals, bioactive compounds with nutritional potential, polyphenolic compounds, polyethylenes, and terpenoids, they are used for cancer prevention and treatment (31-33). Also, the anticancer properties of several plants from this family, such as *Ferulago Mughlea Peşmen*, *Seseli petraeum*, and *Petroselinum crispum*, have been investigated in other research studies (33-35). This is the first study of the anticancer and apoptosis-inducing properties of *F. subpinnata* on cancer.

The GC-MS analysis results of *F. subpinnata* aligned with the study of Bahrami et al. (22). However, compared to other articles, there are some differences (25, 36). It seems possible that these differences are due to various reasons, such as the difference in selected parts of the plant for sampling, the season, and the harvesting area of the plant. The GC-MS showed that the most abundant compounds of this plant have anticancer properties. As mentioned in other articles, compounds like phytol, cinnamaldehyde, and neophytadiene play a role in inducing cell death and preventing cell proliferation (37-39).

In the present research, *F. subpinnata* extract reduced the survival rate of MCF-7 cancer cells and increased intracellular oxidative stress. Many studies have shown that the increased intracellular oxidative stress can induce different models of cell death, including apoptosis (40). Based on observations, it can be concluded that the extract induces cell death by increasing intracellular oxidative stress. This conclusion is supported by the fact that the extract's effect on the survival rate and intracellular oxidative stress subsided when NAC was introduced. Other studies indicated that *F. subpinnata* has relatively good antioxidant properties (26, 41), while in the present research, high concentrations of *F. subpinnata* increased the ROS levels when treating MCF-7 cells. This raises questions about *F. subpinnata* discrepancy, which will be discussed in the following. Even though the extract showed an antioxidant effect in MCF-7 cancer cells in sub-toxic concentrations, the effect of the extract was pro-oxidant in doses that significantly decreased the survival rate. This conflicting effect has been reported in different studies of various herbal compounds. Studies have shown that plant extracts and compounds derived from them have an antioxidant effect (42, 43), yet these compounds can induce cell death and stimulate apoptosis by increasing ROS levels in cancer cell lines (44-46). As Lambert and Elias demonstrated in their study, catechins are chemical antioxidants that can quench free radical species; however, they are also related to the induction of oxidative stress in cancer cells (47). Similarly, acyl pelargonidin derivatives observed in red radishes have antioxidant and pro-oxidant properties (48). The induction of apoptosis in tumor cells can result from pro-oxidant effects (47, 49). Increasing intracellular oxidative stress by herbal compounds induced apoptosis in cancer cell lines, so it could be concluded that the extract of *F. subpinnata* could induce apoptosis in the MCF-7 cancer cell line. In another way, this discrepancy can be well described based on GC-MS analysis. In this regard, phytol and MEHP could increase the ROS levels while other antioxidative compounds, such as cinnamaldehyde, could decrease the ROS levels. Studies showed cinnamaldehyde was effective in significantly decreasing the levels of ROS in arthritic rats (50). On the other hand, MEHP caused strongly amplified production of ROS and activation of *p53* and *p21* in the LNCaP human prostate adenocarcinoma cell line (51). Similarly, another study explained the apoptotic property of phytol by increasing ROS through the induction of caspase 9 and 3 in the lung carcinoma cell line (A549) (52).

Apoptosis is one of the important processes in cells, with two internal and external pathways. The intrinsic pathway inside the mitochondria is caused by various extra and intracellular stresses, as well as oxidative stress, ultimately causing cell death. The balance between anti-apoptotic proteins and pro-apoptotic proteins regulates cell death. Anti-apoptotic proteins *Bcl-2* and *Bcl-xL* (member of the *Bcl-2* family) prevent the release of cytochrome c; on the other hand, pro-apoptotic proteins such as *BAX* provoke the release of Cyt-c by activating the caspase CASP9 pathway (48, 53). Our results indicated that the extract could induce apoptosis through the internal apoptosis pathway by modulating *BAX* and *BCL-2* expressions. These changes aligned with an increase in ROS level and *TP53* expression. Various studies have shown that the Apiaceae family has pro-apoptotic and antiproliferative effects on different cancer cell lines (54, 55).

### 5.1. Conclusions

This study determined the anticancer properties of *F. subpinnata* extract for the first time. The results showed that the extract of this plant increased the level of intracellular oxidative stress and induced apoptosis in MCF-7 cancer cells. It is suggested to investigate the effect of this plant extract on cancer in animal models.
